# Supplementary material for: Evaluating the Potential of Large Language Models for Vestibular Rehabilitation Education: A Comparison of ChatGPT, Google Gemini, and Clinicians
Source: Phys Ther. 2025 Feb 11;105(4):pzaf010. doi: 10.1093/ptj/pzaf010 (PMC11994992; doi:10.1093/ptj/pzaf010)
Supplement: 2024-0323_R1_Supplementary_Material_pzaf010 [file 2024-0323_r1_supplementary_material_pzaf010.pdf]

| Vestibular Knowledge Test – VKT |
| --- |
| Which of the following statements is true in the context of the vestibular system's function?<br>A. The vestibular system works only during movement. Excitation and inhibition are generated depending on the movement<br>**B. The vestibular system works all the time, even when we are not in motion. Excitation and inhibition are generated according to the direction of motion**<br>C. The vestibular system transmits information to the brainstem through the optic nerve<br>D. The vestibular system is not active when fixation is disabled (in the dark or with Frenzel glasses) |
| Which of the following statements is true regarding oculomotor eye movements?<br>A. Oculomotor eye movements are in the opposite direction to the head's movement<br>**B. Oculomotor eye movements enable gaze stabilization during slow movement or without head movement**<br>C. Oculomotor eye movements are produced when the head moves at a high frequency<br>D. Oculomotor eye movements are reflexes and generated in the vestibular organ |
| In acute vestibular syndrome of the right ear, according to Alexander's law, what should we expect to see in the first few hours and days?<br>A. Nystagmus increases when the gaze is diverted to the right side and nystagmus decreases when the gaze is diverted to the left<br>B. Nystagmus increases following visual fixation on an object, nystagmus decreases when the ability to visually fixate is barred<br>C. Nystagmus increases when the gaze is directed forward, nystagmus decreases when the gaze is diverted to the sides<br>**D. Nystagmus increases when the gaze is diverted to the left side and nystagmus decreases when the gaze is diverted to the right** |
| In acute vestibular syndrome of the left ear, according to Alexander's law, what should we expect to see in the first few hours and days?<br>**A. Nystagmus increases when the gaze is diverted to the right side, and nystagmus decreases when the gaze is diverted to the left**<br>B. Nystagmus increases when the gaze is diverted to the left side, and nystagmus decreases when the gaze is diverted to the right<br>c. Nystagmus increases with visual fixation on an object, nystagmus decreases when the ability to visually fixate is barred<br>d. Nystagmus weakens when the ability to visually fixate is barred |
| When interviewing a dizzy patient, what will be the questions' main focus?<br>A. Characterization of the functional impairments of the patient at home and work<br>B. Assessing the patient's imaging and laboratory test results from previous treatments<br>**C. Characterizing the patient's symptoms – a description of the dizziness, its duration and the circumstances of its appearance**<br>D. Evaluation of musculoskeletal disorders with an emphasis on neck problems |
| A patient reports that he has been suffering from dizziness for over a week, what will you never overlook when interviewing the patient?<br>A. An ADL function test with an emphasis on changing positions and turning in the bed<br>**B. A description of how the patient feels, the duration of the dizziness, and the circumstances of its appearance** |

| |
|---|
| C. Obtaining information about the patient's physical activity<br>D. Inspection of imaging tests he performed due to the dizziness |
| In which of the following tests Frenzel glasses must be used while examining a dizzy patient?<br>A. In the Head Impulse test, the ability to perform visual fixation must be barred<br>B. In the VOR cancellation test, the ability to perform visual fixation must be barred<br>C. There is no need to use Frenzel glasses to bar visual fixation in any of the tests<br>D. **While preforming a head-shaking nystagmus test, that ability to perform visual fixation must be barred** |
| Which of the following examinations is used to diagnose the vestibular system?<br>A. **Head Impulse test**<br>B. Saccades test<br>C. Smooth Pursuit test<br>D. VOR cancellation |
| When examining a dizzy patient, which of the following tests is different in terms of the meaning of the result that is obtained?<br>A. Head Impulse test<br>B. Spontaneous nystagmus without fixation<br>C. **VOR cancellation**<br>D. Head-shaking nystagmus |
| Which of the following statements is correct in regard to a vestibular test?<br>A. A vestibular test must include only tests that enable visual fixation<br>B. A vestibular test must only be conducted without the ability to fixate<br>C. **A vestibular test must include tests with and without visual fixation**<br>D. None of the above |
| While performing Dix-Hallpike tests to the right and left, the patient experiences vertigo in both tested positions. You observed a geotropic horizontal nystagmus in each test, how will you proceed in order to reach a diagnosis?<br>A. I will start treating the ear with the more severe nystagmus, and afterwards, or in the next session, I will treat the other ear<br>B**. I will proceed to the Roll test and assign treatment according to the direction of the nystagmus and its intensity**<br>C. As the observed nystagmus is not typical to BPPV problems, I will refer the patient to a neurologist<br>D. As the observed nystagmus is not typical to BPPV problems, I will perform a VBI to rule out red flags |
| While performeing the Dix-Hallpike tests to the right and to the left, you observed an up-beating rotatory nystagmus with a rotation to the left in both sides. What do you think the patient is suffering from?<br>A. BPPV on both sides<br>B. Left horizontal canal BPPV<br>C. The patient does not suffer from a vestibular problem<br>D. **BPPV left posterior canal** |
| One month after being diagnosed with inflammation of the vestibular nerve, a patient complains of gaze instability and lack of balance. On what will your training focus?<br>A. **Adaptation and balance exercises** |

B. Habituation and adaptation exercises
C. Balance and substitution exercises
D. Habituation and substitution exercises

What must be included in adaptation training of the vestibular system?
A. Practice with closed eyes
B. **Head movements during gaze fixation**
C. Static standing on a narrow base
D. Positional therapy

You receive a patient with left posterior canalithiasis BPPV and perform an Epley maneuver. What will you do after completing the maneuver?
A. I will send the patient home and ask him to come again in two days for a re-evaluation, because the patient should not lie down again in the examination position
B. **I will repeat the examination; if the signs and symptoms persist, I will repeat the maneuver and stay a little longer in each position or switch to the Semont maneuver**
C. I will repeat the examination; if his symptoms persist, I will repeat the maneuver and accompany the maneuver with tapping on the treated ear
D. I will repeat the examination; if the signs and symptoms persist, it is likely that I misdiagnosed the involved canal and will, therefore, perform the maneuver on another canal

While performing a Dix-Hallpike tests to the right and to the left, a down-beating nystagmus with a rotation to the right appears in both tests. What treatment will you choose to perform?
A. Postural treatment for the anterior canal by performing an Epley maneuver to the left side (rolling from the healthy side to the involved side)
B. A positioning treatment for the posterior canal by performing a BBQ maneuver
C. A positioning treatment for the right posterior channel by performing a Gufoni maneuver – laying the patient on his involved side and turning his head to the ceiling
**D. A positioning treatment for the anterior canal by performing an Epley maneuver to the right side (rolling from the involved side to the healthy side)**

in which findings you must immediately refer the patient to the emergency room?
A. Pure rotary or vertical nystagmus
B. Decreased hearing during a dizziness episode
C. **Positive HINTS test**
D. All of the above

While performing oculomotor tests, you observe a vertical deviation during the Cover Uncover test, a changing-direction nystagmus when the gaze is held on both sides, and during the Head Impulse test - no noted vestibular impairment. Given these findings, what should you do?
A. **Refer the patient urgently to the emergency room**
B. Refer the patient to his General practitioner doctor for further diagnosis
C. Complete the test and guide the patient to perform habituation exercises
D. Refer the patient to a cardiology consultation

After you successfully treat a patient diagnosed with BPPV, what will be your post-treatment recommendations?
A. Avoid extending or flexing the head for 48 hours, by using a spongy collar

| |
|---|
| B. Make sure to sleep with the head in an elevated position by using pillows or by sleeping in a semi-sitting position<br>**C. Return to normal activity; you must not refrain from movement or restrict the sleeping position but should avoid climbing ladders or stools**<br>D. All of the above |
| After you successfully treat a patient with BPPV, what would you recommend the patient to avoid doing at home?<br>A. Avoid bending down or raising the head<br>B. Avoid lying on the side that was treated<br>C. Avoid removing the neck collar for 24–48 hours<br>D**. Avoid climbing a ladder** |
